# The MDM2 ligand Nutlin-3 differentially alters expression of the immune blockade receptors PD-L1 and CD276

**DOI:** 10.1186/s11658-020-00233-w

**Published:** 2020-08-31

**Authors:** Ruidong Li, Pavlina Zatloukalova, Petr Muller, Maria Gil-Mir, Sachin Kote, Simon Wilkinson, Alain J. Kemp, Lenka Hernychova, Yaxin Wang, Kathryn L. Ball, Kaixiong Tao, Ted Hupp, Borivoj Vojtesek

**Affiliations:** 1 University of Edinburgh, Institute of Genetics and Molecular Medicine, Edinburgh, Scotland EH4 2XR UK; 2 Department of Gastrointestinal Surgery, Union Hospital, Tongji Medical College, Huazhong University of Science and Technology, Wuhan, China; 3 RECAMO, Masaryk Memorial Cancer Institute, 656 53 Brno, Czech Republic; 4 University of Gdansk, International Centre for Cancer Vaccine Science, ul. Wita Stwosza 63, 80-308 Gdansk, Poland; 5 Department of Anesthesiology and Critical Care, Union Hospital, Tongji Medical College, Huazhong University of Science and Technology, Wuhan, China

**Keywords:** MDM2, p53, Gene editing, Protein-protein interactions, Nutlin-3

## Abstract

**Background:**

The links between the p53/MDM2 pathway and the expression of pro-oncogenic immune inhibitory receptors in tumor cells are undefined. In this report, we evaluate whether there is p53 and/or MDM2 dependence in the expression of two key immune receptors, CD276 and PD-L1.

**Methods:**

Proximity ligation assays were used to quantify protein-protein interactions in situ in response to Nutlin-3. A panel of p53-null melanoma cells was created using CRISPR-Cas9 guide RNA mediated genetic ablation. Flow cytometric analyses were used to assess the impact of *TP53* or *ATG5* gene ablation, as well as the effects of Nutlin-3 and an ATM inhibitor on cell surface PD-L1 and CD276. Targeted siRNA was used to deplete CD276 to assess changes in cell cycle parameters by flow cytometry. A T-cell proliferation assay was used to assess activity of CD4+ T-cells as a function of *ATG5* genotype.

**Results:**

CD276 forms protein-protein interactions with MDM2 in response to Nutlin-3, similar to the known MDM2 interactors p53 and HSP70. Isogenic HCT116 p53-wt/null cancer cells demonstrated that CD276 is induced on the cell surface by Nutlin-3 in a p53-dependent manner. PD-L1 was also unexpectedly induced by Nutlin-3, but PD-L1 does not bind MDM2. The ATM inhibitor KU55993 reduced the levels of PD-L1 under conditions where Nutlin-3 induces PD-L1, indicating that MDM2 and ATM have opposing effects on PD-L1 steady-state levels. PD-L1 is also up-regulated in response to genetic ablation of *TP53* in A375 melanoma cell clones under conditions in which CD276 remains unaffected. A549 cells with a deletion in the *ATG5* gene up-regulated only PD-L1, further indicating that PD-L1 and CD276 are under distinct genetic control.

**Conclusion:**

Genetic inactivation of *TP53*, or the use of the MDM2 ligand Nutlin-3, alters the expression of the immune blockade receptors PD-L1 and CD276. The biological function of elevated CD276 is to promote altered cell cycle progression in response to Nutlin-3, whilst the major effect of elevated PD-L1 is T-cell suppression. These data indicate that *TP53* gene status, ATM and MDM2 influence PD-L1 and CD276 paralogs on the cell surface. These data have implications for the use of drugs that target the p53 pathway as modifiers of immune checkpoint receptor expression.

## Introduction

Mouse double minute 2 homolog (MDM2) is a RING domain containing E3-ubiquitin ligase that regulates the function of the p53 tumor suppressor in normal cells through a positive feedback loop [1]. The *MDM2* gene is also amplified or stimulated transcriptionally in human cancers, resulting in attenuation of the tumor suppressor function of p53 [2]. Inhibition of p53 function by MDM2 can occur either through suppression of p53-dependent transcription [3] and/or by stimulating p53 protein ubiquitin-dependent degradation [4]. The primary interface through which MDM2 binds p53 occurs between a deep hydrophobic peptide-binding groove on MDM2 that interacts with the transactivation domain of p53 [5]. Peptides derived from p53 can compete with MDM2 binding to p53 [6], resulting in p53 transactivation and providing the proof-of-concept that MDM2 protein is druggable [7]. Peptide-mimetic drugs named Nutlins that activate p53 function were developed [8]. There are now numerous MDM2 drug leads that target this N-terminal hydrophobic pocket in preclinical or clinical trials [9–11]. Understanding the mode of action of this class of MDM2 targeted drugs will be important as patients with diverse cancer types begin to be treated with MDM2 inhibitors.

Human sarcomas with *MDM2* gene amplification were the first cancer types to be trialled with MDM2 targeted drugs [12, 13]. In the first clinical trial, more than half of the patients tested exhibited stable disease with only one patient showing remission [14]. These data suggested that tumors could indeed respond to MDM2 drugs, by ‘activating’ p53, but the absence of complete regression in the majority of patients casts some doubt on whether the correct biomarkers were used to stratify patients. The main biomarkers to stratify patients were *MDM2* gene amplification and wild-type*TP53* gene status, and the responding biomarkers were induction of the classic p53-dependent transcription targets. It is now proposed that this class of drug can have an “on-target toxicity” [15]. However, due to the allosteric and agonist effect of Nutlin-3 on MDM2 protein, the molecule can also alter MDM2’s protein-protein interactions [16]. Nutlin-3 functions as an agonist promoting a lower affinity but physiologically significant interaction between the core DNA-binding domain of p53 and the central acidic domain of MDM2 [16, 17]. Novel small molecule screening platforms have also identified an interaction between MDM2 and the central domain of p53 [18]. It is this second interaction between p53 and its oncogenic E3-ligase partner that constitutes a ‘ubiquitination signal’ for p53. Nutlin-3 can also stabilize a protein complex between p53, MDM2 and MDMX [19]. In cells, Nutlin-3 can stimulate MDM2 binding to p53 in the nucleus and catalyze a monoubiquitination event(s) that stabilizes p53 binding to chromatin [20]. How Nutlin-3 impacts on MDM2 conformation is not well defined using structural biology. However, a variety of methods reveal that peptides or ligands like Nutlin-3 can alter MDM2-N-terminal domain conformation [21–23].

Similar to the MDM2-catalyzed induction of p53 monoubiquitination [20], the pro-oncogenic protein Notch has been shown to be monoubiquitinated and activated by MDM2 using the same “dual-site” ubiquitination mechanism as MDM2 uses for p53 [24]. Nutlin-3 can also promote the de-oligomerization of MDM2-nucleophosmin (NPM) interaction [25], providing additional evidence for an agonist effect of Nutlin-3 on MDM2 by re-wiring MDM2’s protein-protein interactions. The Nutlin-3 responsive proteins can be stratified with respect to p53-like “BOX-I” homology motifs that identify a relatively large set of p53-like MDM2 binding proteins whose equilibrium binding to MDM2 is disturbed by Nutlin-3 [26]. Mass spectrometry demonstrated that mitochondrial protein interactions are perturbed by Nutlin-3, which induces an increase of MDM2 binding to the dihydrolipoamide dehydrogenase (DLD) subunit of mitochondrial pyruvate dehydrogenase in the nucleus [27]. Thus, the balance between tumor suppressor induction and oncoprotein activation by MDM2-targeted drugs could tip the balance towards tumor regression or tumor survival, respectively.

Because the first clinical trial with an MDM2 targeted drug lead did not result in significant tumor regression despite p53 biomarkers being activated in vivo [14], a question arises as to whether the tumors escape immune-mediated eradication despite p53 activation. The cancer field has only recently begun to link cancer genes to immune system functions. For example, Myc has been shown to up-regulate tumor PD-L1 expression, thus providing a direct link between cancer cell proliferation and anti-tumor immunity [28]. However, by contrast, another study has shown that Myc can also decrease tumor intrinsic PD-L1 expression in the context of interferon stimulation [29], highlighting the complexity of Myc in regulating cancer-immune cell interactions. Oncogenic Ras can also promote tumor immunoresistance by stabilizing PD-L1 mRNA [30]. There have been very few studies examining the impact of p53 on PD-L1 expression. One report published during the course of this current study indicated that the p53 activating molecule APG-115 can promote antitumor immunity in the tumor microenvironment regardless of the p53 status of tumors [31].

In this report, we begin to define fundamental links between p53 pathway functionality and PD-L1 family expression. We evaluated the expression of two key immune checkpoint receptor paralogs, programmed death-ligand 1 (PD-L1/CD274/B7-H1) and its closest ortholog, cluster of differentiation 276 (CD276/B7-H3), as a function of p53 gene status and p53 regulatory molecules. We show for the first time that these receptors can be regulated by drug treatments that target MDM2 or ATM, Nutlin-3 and KU5593, respectively. We have also determined a p53 dependence in CD276 or PD-L1 protein expression that has implications for how MDM2-targeted drugs can impact on immune blockade receptor superfamily expression. These data also highlight that MDM2-targeted drugs impact on the cell surfaceome and further expand the MDM2 protein-protein interaction landscape.

## Materials and methods

### Cell culture and reagents

A375 (ATCC CRL-1619) cells and *TP53*-null derivatives of A375 (generated in this study), and A549 (ATCC CCL-185) and *ATG5*-null A549 derivative cells [32] were grown in DMEM (Sigma-Aldrich). HCT116 (ATCC CCL-247) cells (p53-wt and p53-null cells [33]) were grown in McCoy’s 5A Medium (Sigma-Aldrich). Both media contained 10% fetal bovine serum (FBS), 1% pyruvate and penicillin/streptomycin and all cell lines were incubated at 37 °C in a humidified atmosphere with 5% CO_2_. Cells were grown to 60–80% confluence prior to treatment with Nutlin-3 (Sigma-Aldrich), KU55993 and MG-132 (both Selleckchem).

### Western blotting

Sodium dodecyl sulphate-polyacrylamide gel electrophoresis and immunoblotting were performed as described previously [34]. Primary antibodies against PD-L1 (E1L3N) XP (Cell Signaling Technology), CD276 (B7-H3) (R&D Systems Inc.), MDM2 (4B2) [35], p53 (DO-1) [36], β-actin and GAPDH (both Santa Cruz Biotechnology) were used. Secondary antibodies were HRP-conjugated swine anti-rabbit, HRP-conjugated rabbit anti-mouse (both Agilent) and HRP-conjugated donkey anti-goat (Abcam).

### Proximity ligation assays

A375 cells were grown in 24-well plates over glass coverslips. Cells were treated with DMSO or 10 μM Nutlin-3 when cell confluence reached approximately 50%. Cells were fixed with 4% paraformaldehyde for 15 min, permeabilized using 0.25% TritonX-100 for 10 min, and blocked in 3% bovine serum albumin (BSA) for 30 min. Cells were incubated with combinations of mouse anti-MDM2 (4B2) and polyclonal goat CD276 (B7-H3), rabbit anti-p53 (CM-1) [37] or rabbit anti-HSP70 (SPA812, Enzo Lifesciences) overnight at 4 °C. After removal of unbound primary antibodies, cells were incubated with proximity probes (anti-goat or anti-rabbit PLUS and anti-mouse MINUS) (Duolink, Merck) for 1 h at 37 °C. The hybridization step was performed for 30 min at 37 °C, followed by ligation for 30 min at 37 °C and polymerization reaction for 2 h at 37 °C. The cells were counterstained with DAPI. Coverslips were mounted with Vectashield and captured using an Olympus BX51 microscope (Olympus).

### Flow cytometric analysis

In order to measure expression of receptors on the cell surface, tumor cells were incubated with DMSO, Nutlin-3 or KU55993 for 24 h. The cells were harvested, washed twice with phosphate buffered saline (PBS), resuspended in PBS containing fluorescein isothiocyanate (FITC)-conjugated anti-CD276 (Miltenyi Biotec), allophycocyanin (APC)-conjugated anti-PD-L1 (R&D Systems Inc.) or isotype controls and incubated in the dark for 30 min at 4 °C. Then cells were washed and CD276 or PD-L1 on the cell surface was measured by the fluorescent properties of antibodies in the channel for FITC (Ex-Max 494 nm/Em-Max 520 nm) and for APC (Ex-Max 650 nm/Em-Max 660 nm). The fluorescent signals were evaluated by BD Accuri C6 (BD Biosciences) using CFlow Plus software (BD Bioscience). Data were analyzed in triplicate by FCS Express version 3.0 (De Novo Software).

### p53 knockout using the CRISPR-CAS9 system

The *TP53*-specific sgRNA sequence was 5′-CTGAGCAGCGCTCATGGTGGNGG-3′ and was cloned into the pBT-U6-CAS9-2A-GFP expression vector to create pBT-U6-CAS9-2A-gp53-GFP. *TP53* gene editing in the A375 (p53-wt) melanoma cell line was performed as described before with minor alterations [38]. Briefly, A-375 cells were seeded in 6-well plates using 300,000 cells per well and left to adhere for 24 h. Then cells were transfected with pBT-U6-CAS9-2A-gp53-GFP using Attractene transfection reagent (Qiagen). After 48 h, mutations were tested using a Surveyor mutation detection kit (IDT) and GFP-positive cells were sorted and collected by BD FACSCanto II (BD Biosciences), and seeded in 96-well plates (1 cell per well). After 2 weeks, all colonies were collected and tested by Western blotting and sequenced to confirm the *TP53* gene mutation. Functionality of the p53 pathway was tested using an X-irradiation source (Faxitron Bioptics, LCC; 5 Gy at 2.5 Gy/min for 2 min). Functional pathway ablation was confirmed by assessing p53 and p21^WAF1^ protein induction after 4 h post-irradiation (Supplementary Fig. 1).

### siRNA and cell cycle analysis

One million cells per sample were used for transfection with 50 nM *CD276* specific silencing RNA (Dharmacon) by Amaxa nucleofector technology (Lonza). After 48 h, the cells were exposed to 20 μM Nutlin-3 or DMSO (at a final concentration of 0.05% DMSO) for 48 h. Cells were harvested, fixed in 70% ethanol for 2 h on ice, washed in PBS, resuspended in staining solution containing 0.1% Triton X-100, RNase A and propidium iodide at final concentrations of 100 μg/ml and 5 μg/ml, respectively, and incubated in the dark for 10 min. The samples were analyzed using a BD FACSVerse (BD Biosciences). Data were analyzed in triplicates by FCS Express version 3.0 (De Novo Software).

### T-cell assays

CD4 positive T (CD4+ T) cells were isolated from peripheral mononuclear cells using paramagnetic beads (Stemcell technologies) according to the manufacturer’s instructions. Cell purity was > 90%, which was confirmed by BD LSR II (BD Biosciences). CD4^+^ T cells were labeled using carboxyfluorescein succinimidyl ester (CFSE) (BioLegend) according to the manufacturer’s instructions, cultured in the presence of anti-CD3/CD28 (BD Biosciences) (each at final concentration of 2.5 μg/ml) for 2 days and then incubated with A549-atg5-wt or A549-atg5-null cells at a T-cell: A549 cell ratio of 1:5 for the next 2 days. The samples were analyzed by BD LSR II and the results represent the mean ± SD of technical triplicates, each with 30,000 counted cells.

## Results

### MDM2-CD276 protein-protein interactions in situ

Nutlin-3 has opposite effects on two of the earliest identified MDM2 binding proteins; p53 is stabilized and E2F1 is destabilized [39]. The number of Nutlin-3 induced or destabilized MDM2-binding proteins is not well annotated. Defining the depth of such perturbations might impact on our clinical understanding of how MDM2 targeted drugs show variability in the clinic [14]. In this report, we aimed to determine how the MDM2 ligand Nutlin-3 can impact on immune checkpoint receptor expression. To begin, we first evaluated whether a protein-protein interaction between MDM2 and CD276 or PD-L1 occurs in response to Nutlin-3 treatment. This logic was used considering our prior report highlighting that Nutlin-3 can rewire certain MDM2 protein-protein interactions [27].

**Fig. 1:**
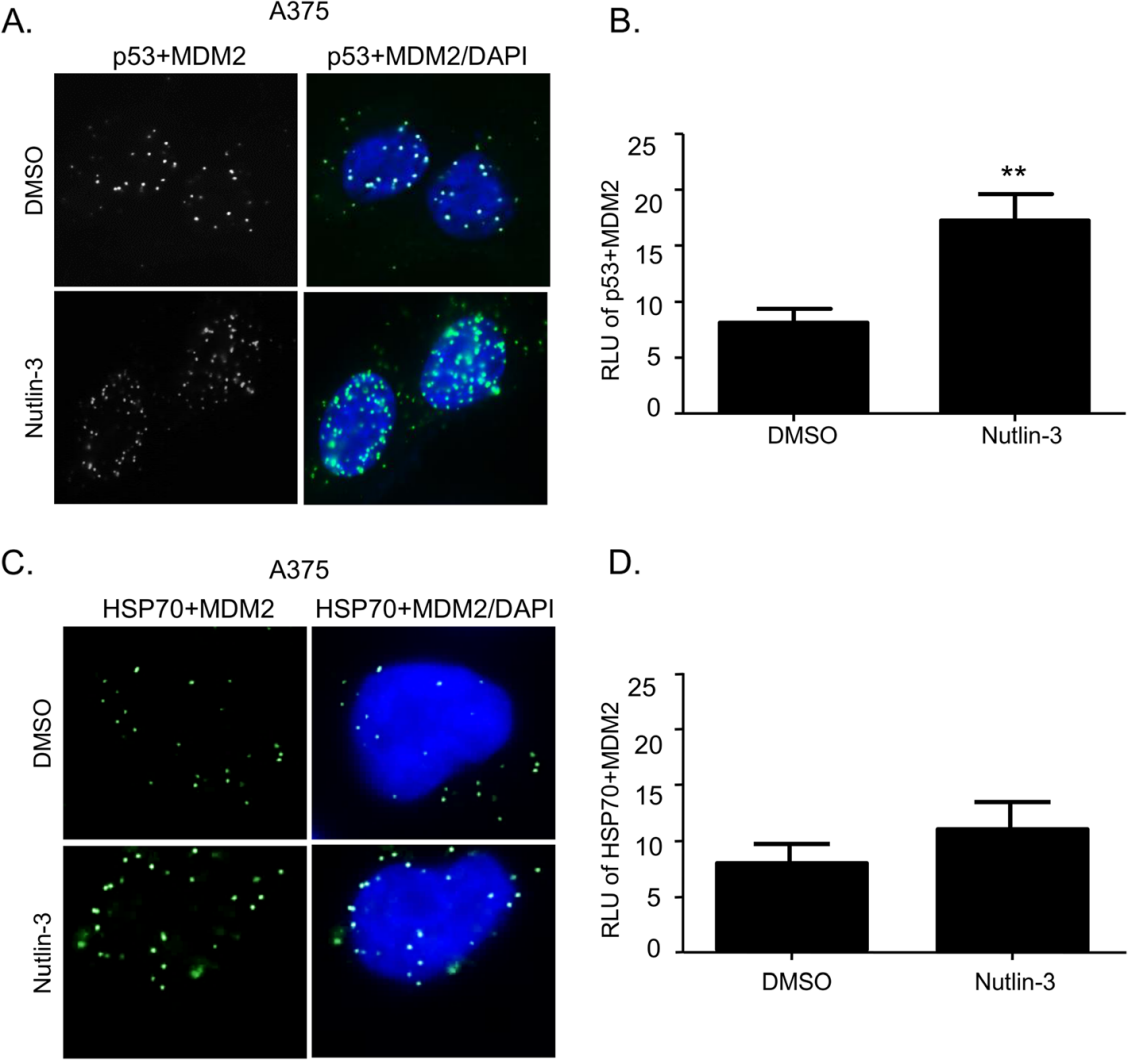
Nutlin-3 stabilizes p53-MDM2 and HSP70-MDM2 protein-protein interactions in situ. **a**, **c** Proximity ligation assay of A375 cells treated with DMSO or 10 μM Nutlin-3 for 24 h. Green fluorescence spots suggest that both target proteins (p53-MDM2 or HSP70-MDM2) are within interacting range. The cells were counterstained with DAPI (blue). **b**, **d** The quantification of p53-MDM2 or HSP70-MDM2 interaction after incubation with DMSO and Nutlin-3. The results represent the mean ± SD of technical triplicates. Statistical analysis was performed by one-way ANOVA with a Bonferroni post hoc test, ** *p* < 0.01

To determine whether CD276-MDM2 or PD-L1-MDM2 complexes are elevated after incubation with Nutlin-3, we used proximity ligation assays (PLA). PLA can be used to identify a protein-protein interaction (or an “association”) in cells, in situ, with a distance of 10–30 nm that is in the upper range of a protein-protein interaction that can be observed using fluorescence resonance energy transfer (FRET) (5–20 nm) [40]. Thus, the advantage of the PLA is that it can detect an authentic endogenous protein-protein complex in situ and it does not rely on artificially constructed, transfected or non-physiologically tagged protein expression vectors. In addition, as lysis disrupts cellular compartmentalization, it is also possible that protein-protein associations present in an immunoprecipitate or other pull-down assay are ‘re-arranged’ post-lysis. First, as controls in setting up this assay, we demonstrate that p53-MDM2 protein-protein interaction foci are induced in the nucleus in response to Nutlin-3 (Fig. 1a and b) as reported previously [20]; this association drives p53 activation through mono-ubiquitination and enhanced chromatin association. In addition, Nutlin-3 can also induce the formation of heat shock protein 70 (HSP70)-MDM2 foci (Fig. 1c and d), consistent with a recent report that demonstrated induction of MDM2 and HSP70 in blood cancer cells in response to the Nutlin ligand [41]. However, this latter report did not provide evidence whether MDM2 and HSP70 formed direct associations in cells. HSP70 is a chaperone thought to cooperate with MDM2 protein folding pathways [42, 43].

**Fig. 2:**
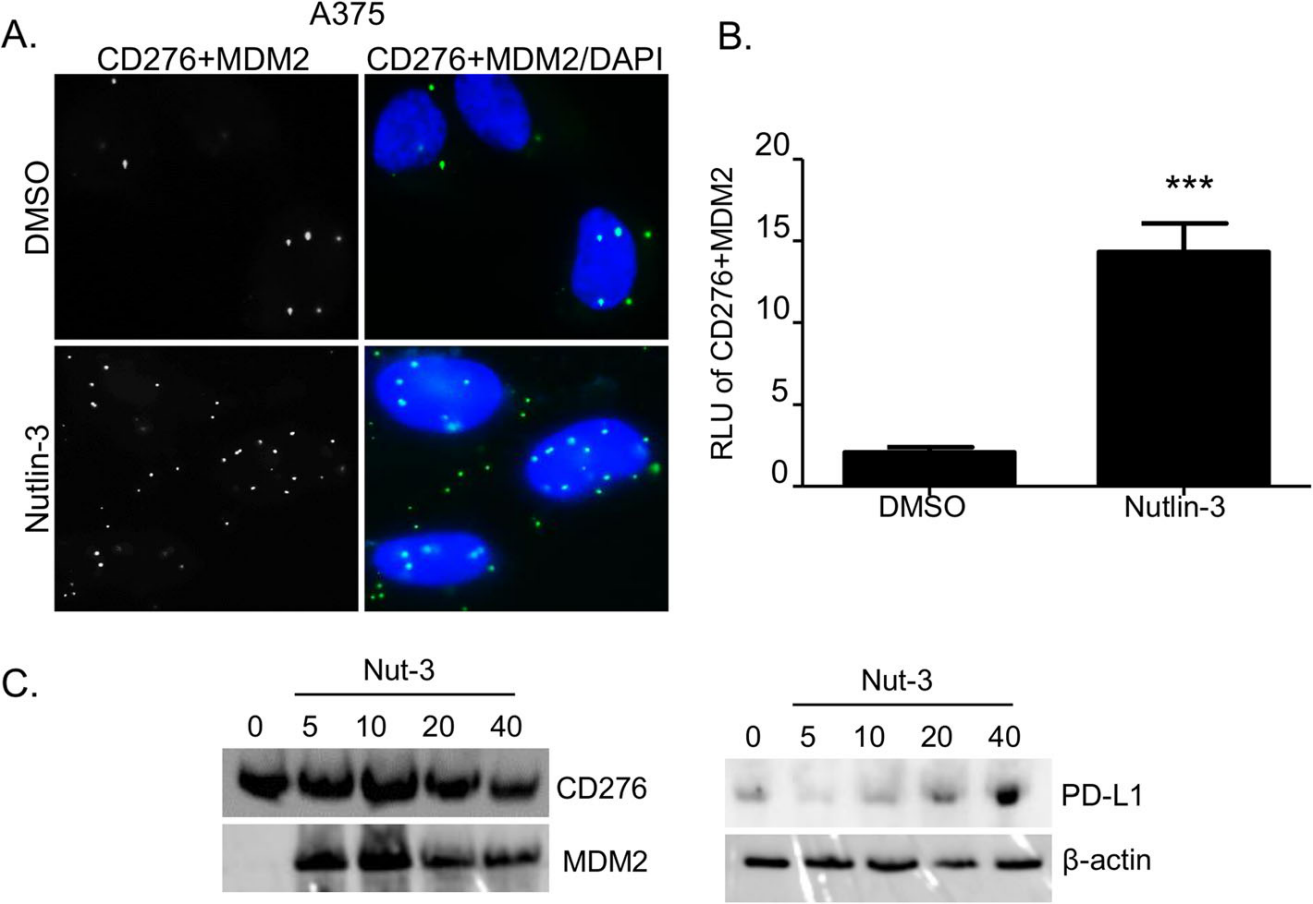
Nutlin-3 stabilizes MDM2-CD276 protein-protein interactions in situ*.* **a** Proximity ligation assay of A375 cells treated with DMSO or 10 μM Nutlin-3 for 24 h. Green fluorescence spots suggest that both target proteins (MDM2-CD276) are within interacting range. The cells were counterstained with DAPI (blue). **b** Quantitation of CD276-MDM2 interaction after incubation with DMSO or Nutlin-3. The results represent the mean ± SD of technical triplicates. Statistical analysis was performed by one-way ANOVA with a Bonferroni post hoc test, *** *p* < 0.001. **c** Western blot of A375 cells treated with the indicated concentrations of Nutlin-3 (Nut-3). MDM2, PD-L1, CD276 and β-actin have apparent molecular weights of 90, 50, 60 and 42 kDa, respectively

With these controls establishing the validity of the PLA upon Nutlin-3 treatment, we evaluated whether CD276 or PD-L1 forms a direct complex with MDM2 in situ in cells and whether this is inhibited or stabilized by Nutlin-3. CD276 and MDM2 foci in growing cells are detectable at a low level and are elevated by Nutlin-3 (Fig.2a and b). These data suggest that there is a direct protein-protein association between these two proteins that is not mediated through a bridging protein, given the distance constraints of the PLA methodology. However, we were not able to detect a complex between PD-L1 and MDM2 in cells using the PLA methodology (data not shown), so we have no evidence that MDM2 can associate with PD-L1. Additionally, total protein levels of MDM2, CD276 and PD-L1 are shown in Fig. 2c.

### Impact of *TP53* gene status on Nutlin-3-responsive cell surface expression of PD-L1 and CD276 receptors

We next evaluated whether the CD276 protein is induced by Nutlin-3, especially whether Nutlin-3 alters the expression of CD276 on the cell surface. It was possible that the enhanced interaction of CD276 and MDM2 induced by Nutlin-3 reflects an intermediate in the CD276 degradation pathway; for example, one impact could be that basal CD276 would actually be suppressed on the cell surface by Nutin-3 treatment.

**Fig. 3:**
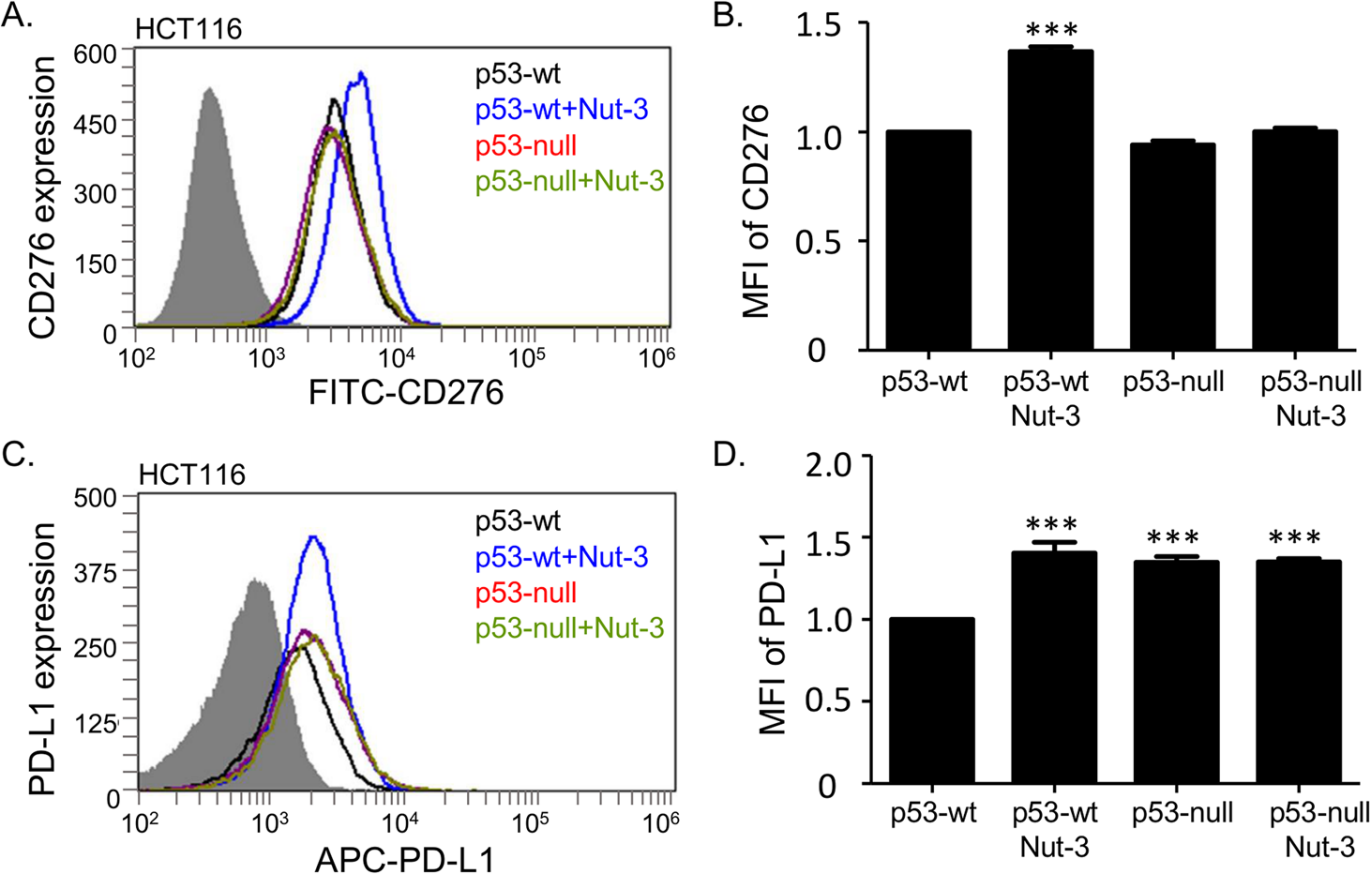
Nutlin-3 induces CD276 and PD-L1 proteins on the surface of HCT116 cells. Representative flow cytometry profiles and quantitation of cell surface CD276 (**a**, **b)** and PD-L1 (**c**, **d**) on HCT116 p53-wt and p53-null cells treated with DMSO or 10 μM Nutlin-3 for 24 h. CD276 and PD-L1 were measured using **a** FITC- and **c** APC-conjugated antibody, respectively. In **a** and **c** the grey areas correspond to an isotype antibody as a negative control. In **b** and **d**, results represent the mean ± SD of technical triplicates, each with 30,000 counted cells. Statistical analysis was performed by one-way ANOVA with Bonferroni post hoc test, *p < 0.05, ***p < 0.001

We evaluated the cell surface expression of CD276 after Nutlin-3 incubation using a fluorescence-activated cell sorting (FACS) based assay, as this is where the presumed fully active forms of CD276 reside [44]. Changes in CD276 on the cell surface were measured in non-permeabilized cells using a FITC-conjugated anti-CD276 antibody [45]. HCT116 cells with p53-wt (HCT116 p53-wt) or lacking p53 (HCT116 p53-null) were used since these cells display p53 dependence in Nutlin-3 induction of MDM2 [8]. The use of the HCT116 cells can also define whether CD276 induction or suppression on the cell surface is p53-dependent, as well as Nutlin-3-dependent. The highest level of CD276 was observed in HCT116 p53-wt cells treated with Nutlin-3 (Fig. 3a and b). These data indicate that Nutlin-3 is able to elevate, rather than reduce, the plasma membrane form of CD276. There was also an apparent p53 dependence in this process, since HCT116 p53-null cells did not display CD276 induction on the cell surface in response to Nutlin-3 (Fig. 3a and b).

We also evaluated the more characterized member of this superfamily, the pro-oncogenic immune blockade target, PD-L1. Surprisingly, cell surface PD-L1 was also induced by Nutlin-3 (Fig. 3c and d) despite the fact that its binding to MDM2 cannot be detected (data not shown). To our knowledge, this is the first observation of Nutlin-3 resulting in upregulation of PD-L1. However, a recent study highlighted that another cancer drug whose activity can be affected by p53 status (5-fluorouracil) also elevates cell surface PD-L1 [46].

### Impact of *TP53* gene status on Nutlin-3-responsive cell surface expression of PD-L1 and CD276 on isogenic A375 p53-wt/null cells

**Fig. 4:**
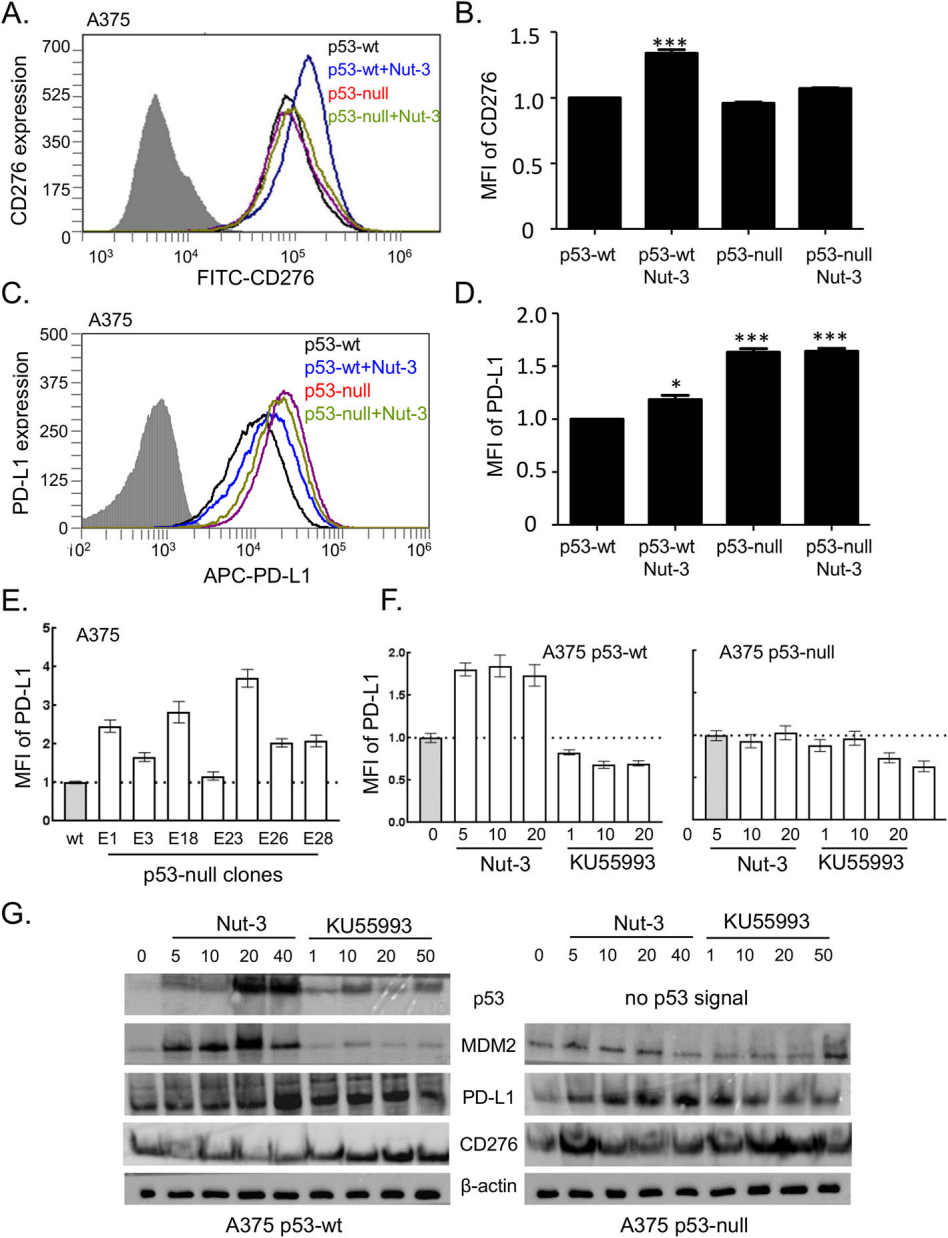
Nutlin-3 induces CD276 and PD-L1 on the surface of melanoma A375 cells. Representative flow cytometry profiles and quantitation of cell surface CD276 (**a**, **b)** and PD-L1 (**c**, **d**) on A375 p53-wt and p53-null cells treated with DMSO or 10 μM Nutlin-3 for 24 h. CD276 and PD-L1 were measured using **a** FITC- and **c** APC-conjugated antibody, respectively. In **a** and **c** the grey areas correspond to an isotype antibody as a negative control. In **b** and **d**, results represent the mean ± SD of technical triplicates, each with 30,000 counted cells. Statistical analysis was performed by one-way ANOVA with Bonferroni post hoc test, *p < 0.05, ***p < 0.001. **e** Measurement of PD-L1 basal level A375 p53-wt (wt) and in single cell-*p53* gene edited clones (Supplementary Fig. 1). The data are plotted as the mean fluorescent intensity (MFI) of PD-L1 for individual clones. **f** The effect of the ATM inhibitory molecule KU55993 and Nutlin-3 (Nut-3) on cell surface PD-L1. A375 p53-wt and A375 p53-null cells were incubated with KU55993 or Nutlin-3 at the indicated concentrations (μM) for 24 h. Western blot of lysates prepared from **g** A375 p53-wt and **h** A375 p53-null cells treated with indicated concentrations of Nutlin-3 (Nut-3) and KU55993. MDM2, p53, CD276, PD-L1 and β-actin have apparent molecular weights of 90, 50, 60, 50 and 42 kDa, respectively

The expression of CD276 in HCT116 cells might be a cell-specific effect and therefore represent an exception. To evaluate the impact of the p53 pathway on CD276 and PD-L1 expression further, we focused on developing a new isogenic A375 p53-wt/null cell panel (for details see Supplementary Fig. 1). We used the A375 melanoma cell line with a wild-type p53 pathway [47–49]. Human melanomas generally maintain wild-type p53 alleles and, as melanoma has a high neoantigen load, it is thought to be a prime tumor type for anti-PD-L1 monoclonal antibody therapies [50]. Using this newly created isogenic A375 melanoma cell panel, we observed a higher level of CD276 on the cell surface in A375 p53-wt cells treated with Nutlin-3 (Fig. 4a and b). PD-L1 exhibited a higher basal level in A375 p53-null cells (Fig. 4c and d). These data are qualitatively similar to the HCT116 (p53-wt and p53-null) cell panel (Fig. 3). To test this hypothesis further, we also analyzed PD-L1 levels in several single cell-gene edited isolates of A375 p53-null cells, thus providing several independent biological replicates. Each of the p53-edited null cells exhibited elevated but variable levels of PD-L1 in the basal state (Fig. 4e). These data using two different cell models (HCT116 and A375) suggest that both PD-L1 and CD276 are under distinct genetic control pathways (e.g. p53 signaling), with a common pharmacological event being their induction by Nutlin-3 in p53-wt containing cancer cells and elevated PD-L1 upon loss of p53-wt protein function.

As another control to test the specificity of Nutlin-3 in the assay which measures induction of PD-L1 in p53-wt cancer cells, we also evaluated the ataxia-telangiectasia mutated (ATM) kinase inhibitor KU55993, which inactivates p53 [51] and therefore has the opposite effect of Nutlin-3 on p53 pathway activity. The ATM inhibitor reduced the basal level of PD-L1 in a dose-dependent manner in p53-wt cells (Fig. 4f) under conditions where Nutlin-3 elevated PD-L1 (Fig. 4f). The reduction of PD-L1 by the ATM inhibitor KU55993 also occurred in the p53-null cell line (Fig. 4f). Additionally, total protein levels of p53, MDM2, CD276 and PD-L1 after treatment with Nutlin-3 and KU55993 on the A375 p53-wt/null cell panel are shown in Fig. 4g.

These data highlight the specificity of Nutlin-3 for induction of PD-L1 and suggest that MDM2 and ATM have opposing effects on PD-L1 steady-state levels. A recent study also highlighted that metformin can suppress PD-L1 in cancer cells [52], and highlights another protein kinase pathway whereby pharmacological targeting can suppress PD-L1. CD276 remained unchanged in assays that measure outer membrane expression after KU55993 treatment (data not shown). These data together suggest that cell surface elevation of PD-L1 by Nutlin-3 or suppression by KU55993 is driven by a different biochemical mechanism than that which regulates the level of cell-surface CD276. In addition, although CD276 is not altered in the basal state by loss of p53, the elevated levels of PD-L1 in mutant p53 cells suggest a mechanism whereby loss of p53 could mediate immune suppression induced by PD-L1.

### Distinct pro-oncogenic functions of CD276 and PD-L1 in cell-based assays

**Fig. 5:**
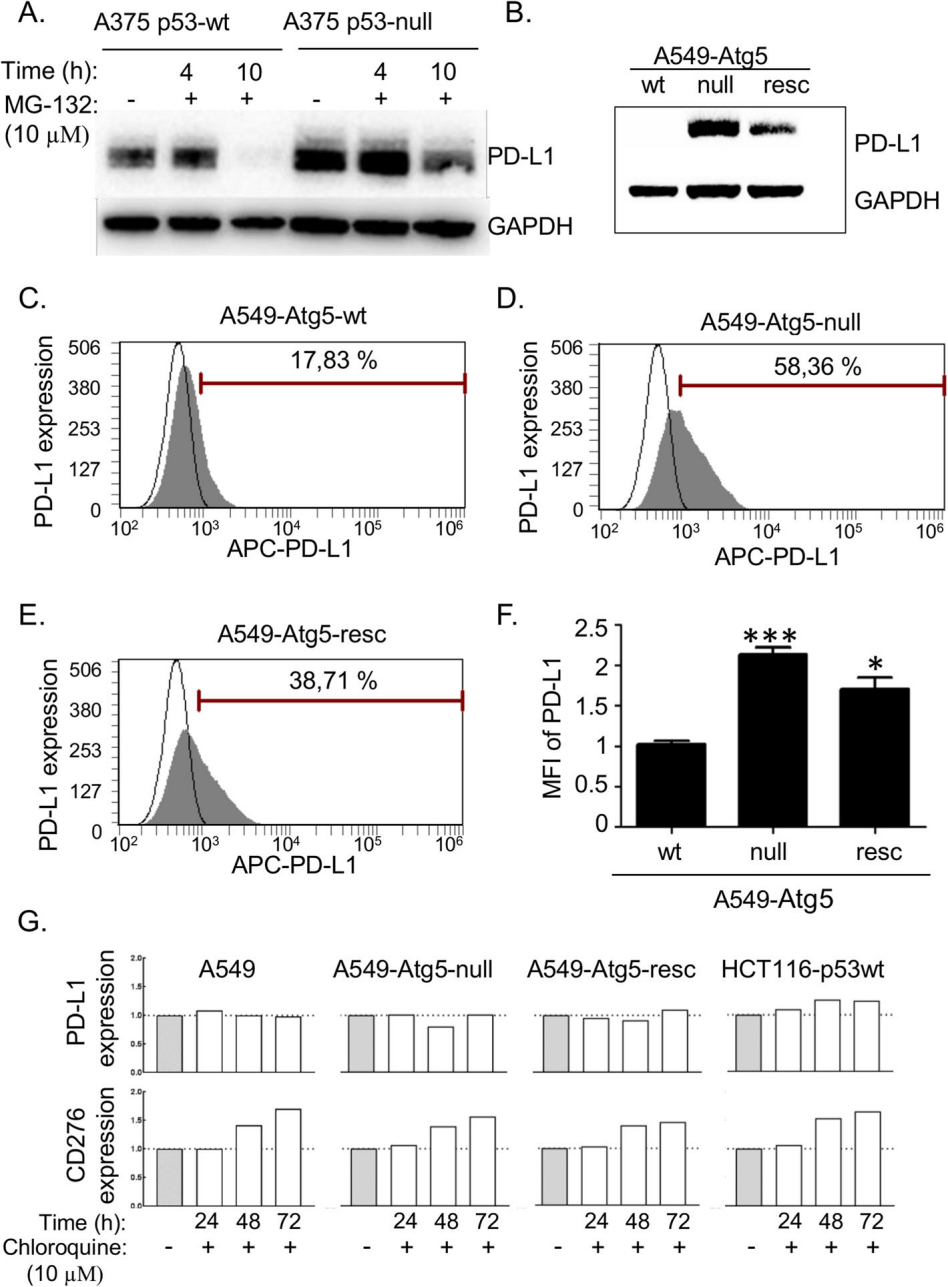
Cell surface PD-L1 as a function of ATG5 status. **a** A375 p53-wt and A375 p53-null cells were treated with 10 μM MG-132 for the indicated times. PD-L1 and glyceraldehyde 3-phosphate dehydrogenase (GAPDH) as a loading control antibody have apparent molecular weights on Western blots of 50 and 37 kDa, respectively. **b** A549-Atg5-wt cells (wt) were processed using gene editing (CRISPR-Cas9) [32] to create mutant A549-Atg5-null cells (null). Atg5-GFP was introduced into A549-Atg5-null cells to create a partial Atg5 rescue phenotype (resc). Both PD-L1 and GAPDH were detected in these cells. Representative flow cytometric graphs of **c** A549-Atg5-wt, **d** A549-Atg5-null and **e** A549-Atg5-resc. Cell surface PD-L1 was measured using APC-conjugated antibody. Results represent the mean ± SD of technical triplicates, each with 30,000 counted cells. **f** Normalized mean fluorescent intensity (MFI) of PD-L1 derived from FACS on **c - e**. Statistical analysis was performed by one-way ANOVA with Bonferroni post hoc test, * p < 0.05, *** p < 0.001. **g** PD-L1 (upper row) and CD276 (lower row) were measured after treatment with 10 μM chloroquine for the indicated times by FACS using APC- and FITC-conjugated antibodies, respectively

Although Nutlin-3 increases cell surface levels of PD-L1, perhaps suggestive of a proteasomal-dependent pathway, we found that the general proteasome inhibitor MG-132 actually reduced steady-state levels of PD-L1 in A375 p53-wt cells (Fig. 5a). This was attenuated in A375 p53-null cells (Fig. 5a), explaining, in part, why PD-L1 is expressed at higher levels in p53-null cells (Fig. 3d and 4d). CD276 was neither induced nor suppressed by MG-132 treatment (data not shown), nor was *CD276* mRNA induced by Nutlin-3 treatment (data not shown). As PD-L1 steady-state levels are reduced by MG-132, we investigated whether PD-L1 and CD276 are again differentiated by virtue of alternate degradation pathways.

Proteins that are not degraded through the proteasome can be processed through the autophagy pathway [53]. Autophagy related 5 (Atg5) protein is used to define autophagy-dependent events in cells [54]. Indeed, Atg5 has recently been reported to regulate PD-L1 levels depending upon cell type [55]. We evaluated CD276 and PD-L1 in adenocarcinoma lung epithelial A549 p53-wt cells containing *ATG5*-wt and mutant *ATG5*-null cells, and in an isolate of A549-Atg5-null cells with re-introduced stably expressed green fluorescent protein (GFP)-Atg5 (Atg5-resc) (Supplementary Fig. 2). PD-L1 protein is elevated in the Atg5-null cells (Fig. 5b). Quantitation of PD-L1 receptor expression on the cell surface was measured in non-permeabilized cells using an APC-conjugated PD-L1 antibody [45]. The data reveal that PD-L1 is significantly elevated in A549-Atg5-null cells and is partially reduced in A549-Atg5-resc cells (Fig. 5c-f). This is consistent with prior evidence demonstrating that Nutlin-3 suppresses p53-dependent autophagy [56]. CD276 was not elevated in Atg5-null cells (data not shown). These data further suggest that the two receptors are regulated by distinct genetic pathways that share common pharmacological control (Fig. 8). CD276 and PD-L1 were measured upon activation of autophagy by treatment with 10 μM chloroquine. PD-L1 expression was not affected by chloroquine at 24 h, 48 h and 72 h after treatment (Fig. 5g, upper row). Induction of CD276 by chloroquine was time-dependent, but this increasing expression may not be related to autophagy because elevation occurred in HCT116 p53-wt, A549-Atg5-wt, A549-Atg5-null and A549-Atg5-rescue cells in the same manner (Fig. 5g, lower row). FACS histograms corresponding to this experiment are included in Supplementary data (Supplementary Fig. 3).

**Fig. 6:**
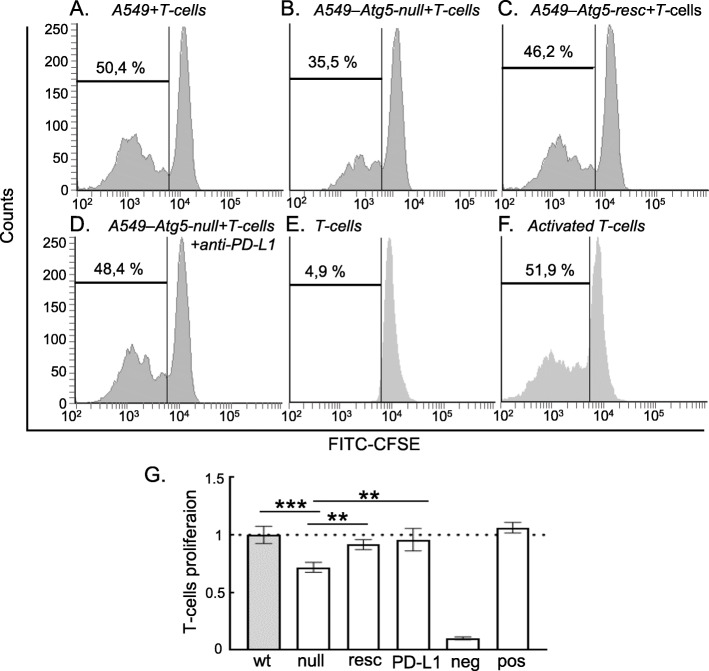
Activity of CD4+ T-cells as a function of ATG5 genotype. Representative flow cytometric histograms with the percentage of proliferating CD4 + T-cells (indicated with black bar) for **a** A549-Atg5-wt, **b** A549-Atg5-null, **c** A549-Atg5-resc, **d** A549-Atg5-null cells in the presence of PD-L1 blocking antibody, **e** only T-cells as negative control and **f** T-cells stimulated with CD/CD28 as positive control. T-cells were labelled with CFSE and co-cultured with the panel of A549 cells at the T-cell: A549 ratio of 1:5. Results represent the mean ± SD of technical triplicates, each with 30,000 counted cells. **g** Normalized quantitative analysis of proliferating T-cells derived from FACS on **a-f**. Statistical analysis was performed by one-way ANOVA with Bonferroni post hoc test, ** p < 0.01, *** p < 0.001

We finally defined the most likely oncogenic function for both CD276 and PD-L1 in cell models. We were not able to evaluate the effects of Nutlin-3 on T-cell responses to the tumor cell lines, since Nutlin-3 induces T-cell death in vitro (data not shown). Instead, we asked whether T-cell activity was suppressed in A549-Atg5-null cells, since these cells express much higher basal PD-L1 (Fig. 5b). In essence, this assay measures whether the elevated PD-L1 is active as an immune suppressant. Relative to A549-Atg5-wt cells, A549-Atg5-null cells exhibited suppression of T-cell proliferation (Fig. 6a, b and e). The stably re-expressed Atg5 in the A549-Atg5-resc cells with reduced PD-L1 levels showed partial restoration of T-cell proliferation functions (Fig. 6c and e). As a further control, PD-L1 blocking antibody is able to also stimulate T-cell proliferation in the A549-Atg5-null cells (Fig. 6d and e). Since CD276 does not change levels in the A549-Atg5-null cells (data not shown), we suggest that the observed T-cell suppression comes primarily from PD-L1 activity. This is consistent with the fact that the PD-L1-blocking antibody restores T-cell functions to wt-levels and that there is no known immune-suppressant function for CD276.

**Fig. 7:**
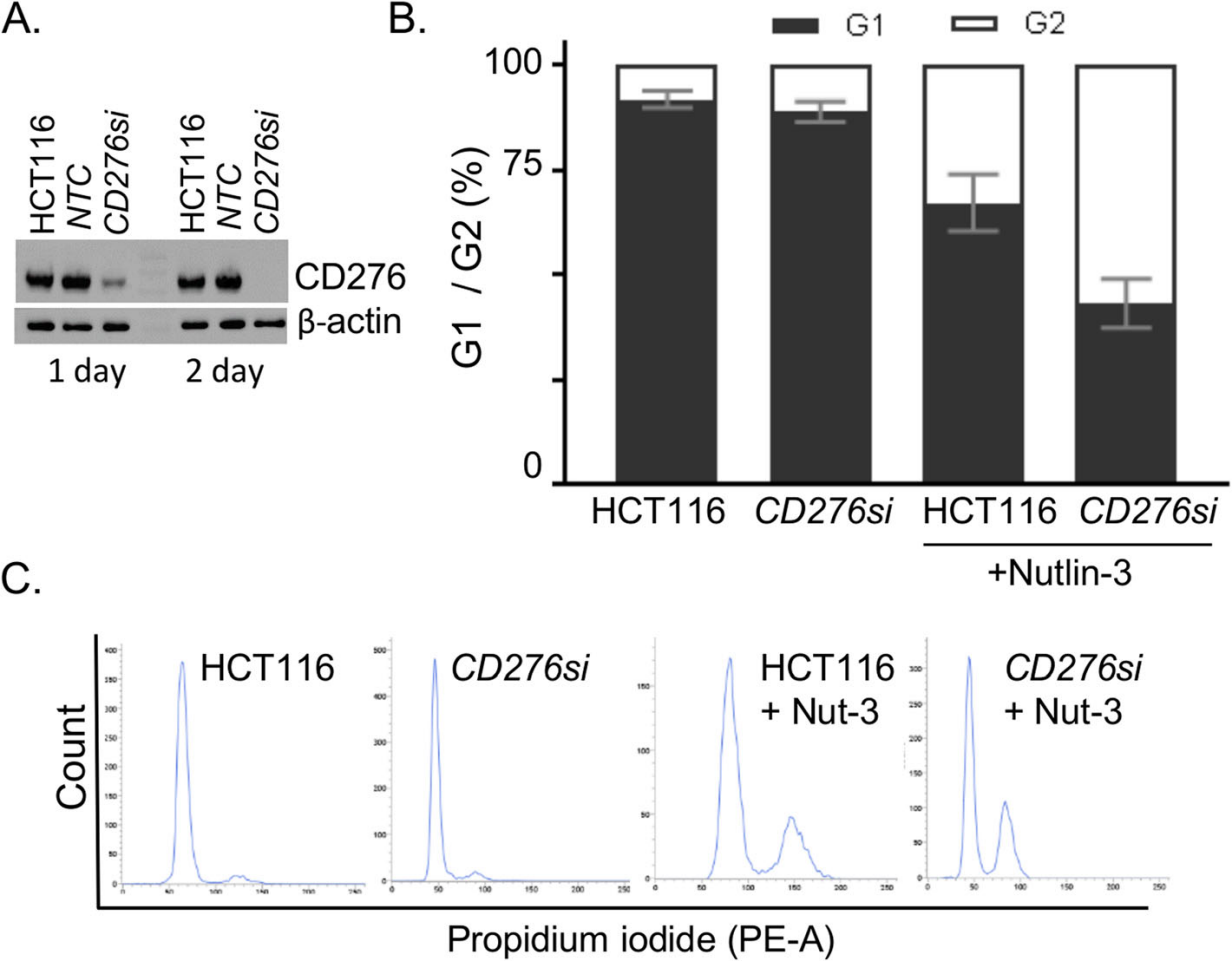
Effects of CD276 depletion on cell cycle parameters. HCT116 p53-wt were transfected with siRNA towards *CD276* (*CD276si*) for 48 h. **a** Detection of CD276 and β-actin on Western blot at molecular weights of 60 and 42 kDa, respectively, *NTC* – non-targeting control. **b** Flow cytometry analysis of cell cycle parameters. Distribution of G1 and G2 phases in HCT116 cells with normal and depleted CD276 protein level (*CD276si*) and after treatment with 20 μM Nutlin-3 or untreated for 48 h. Results represent the mean ± SD of technical triplicates, each with 10,000 counted cells. **c** Representative histograms of cell cycle after staining with propidium iodide

**Fig. 8:**
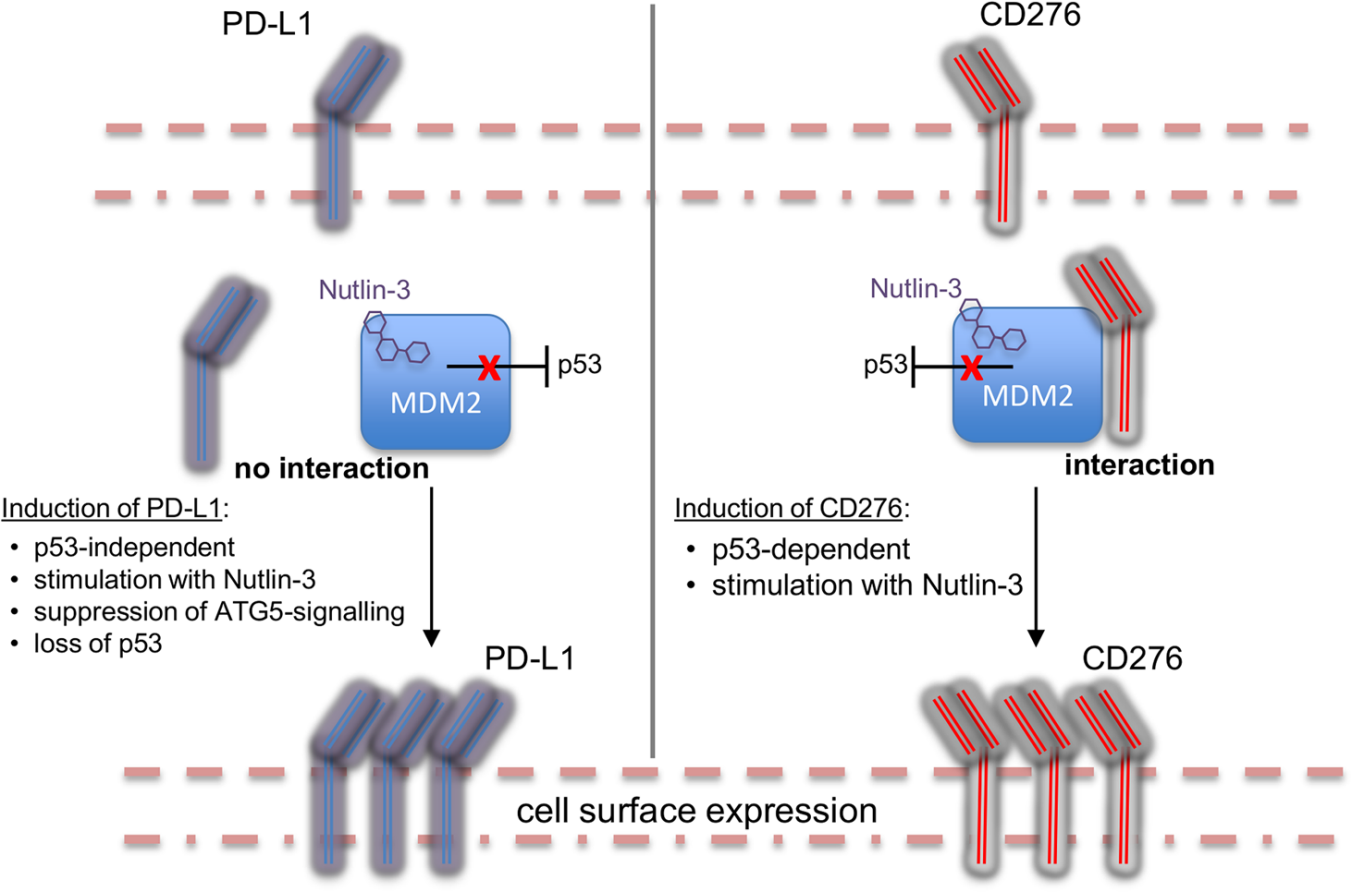
Summary of the genetic and pharmacological events that distinguish regulation and function of CD276 or PD-L1 by the MDM2/p53 axis. The left and right panels summarize data highlighting the elevation of PD-L1 or CD276, respectively, after treatment with Nutlin-3

What might be the function of CD276? Prior reports indicate that CD276 has oncogenic potential [57], although there is no well-validated immune blockade function. Therefore, CD276 protein was depleted using siRNA in HCT116 p53-wt to determine whether any cell cycle phenotypes could be observed to suggest whether or not the protein has intrinsic growth-regulatory roles. The data reveal that reduction of CD276 protein using targeted siRNA (Fig. 7a) has very little impact on cell cycle parameters (Fig. 7b). Hence we determined whether Nutlin-3 effects could potentiate cell growth parameters in this assay. After incubation with Nutlin-3, there is a decrease in the G1 population of cells from 92.8 to 67.1% with an increase in cells in G2 phase from 8.2 to 32.8%. This is consistent with the possibility that Nutlin-3 induces a p53-dependent checkpoint at doses below the threshold of apoptosis. Depletion of CD276 using siRNA potentiated the cell cycle impacts of Nutlin-3, with a further reduction in the number of cells in G1 to 43.2% and a further increase in cells in G2 to 57% (Fig. 7b). These data are consistent with a model suggesting that CD276 may antagonize the effects of Nutlin-3. Together, these data allow us to form a model whereby the *TP53* gene or the *ATG5* gene suppresses PD-L1, whilst their deletion has no impact on CD276. This takes into account data demonstrating that the two receptors are under different genetic control, but with one common pharmacological response, being induction of cell surface receptor expression by Nutlin-3 (Fig. 8).

## Discussion

Despite the fact that MDM2-targeted drugs of the Nutlin class exemplified by RG7112 have a degree of toxicity to patients in the clinic, these drug leads are on target with respect to activation of p53-wt in patient tumors [14]. It is therefore encouraging that a highly specific pharmacological agent can activate p53 function in vivo. This success has encouraged the continued development of novel MDM2-targeted drugs for use in clinical trials (https://www.cancer.gov/about-cancer/treatment/clinical-trials/search). The structures of these novel drug leads are different from the first Nutlin lead RG7112 and include: (i) AMG232 for use in glioblastoma [10]; (ii) the stapled peptide ALRN-6924 that binds to the p53-binding pocket for use in solid tumors or lymphomas [11]; and (iii) RG7388 that is being tested alone and in combination with other chemical agents for use in essential thrombocythemia and polycythemia vera. In our study, we used Nutlin-3 as a robust, cell-membrane-permeable, MDM2-targeted molecule [8] that provides a proof-of-concept for developing assays to define novel pathways that are impacted by MDM2 perturbation.

The on/off rate and/or allosteric effects of these different MDM2-targeted drug leads might impact on MDM2-protein-protein interactions in normal cells and cancer cells. The changes in these protein-protein interaction networks could subsequently lead to MDM2-dependent biomarker discovery for diagnostics or for potentiating therapeutics. For example, the length of the p53 peptide that binds to the MDM2 pocket can alter the shape of MDM2 [58]. This might impact on the MDM2 protein interactome. For example, there is an inverse correlation between the affinity of such peptide ligands for MDM2 and activation *in trans* of MDM2-catalyzed substrate ubiquitination [16]. Hence, we would predict that different MDM2-targeted drugs could have differing signaling impacts that favor p53 activation [8, 20] and NPM de-oligomerization [25] over induction of proteins such as Notch [24], PD-L1 and CD276 (this study, see Supplementary Table 1). The discovery of cellular targets that are regulated by MDM2 activity might lead to improved combinatorial strategies that potentiate the anti-tumor effects of Nutlin-3. For example, we could expect that PD-L1 induction of Nutlin-3 in cancer cells containing p53-wt can promote the suppression of tumor immune tolerance via p53 activation. Combined treatment of such patients with Nutlin-3 and PD-L1 or PD-1 immune blockade antibodies could contribute to enhancing tumor regression.

In the current study, we did not use clinically derived primary cancer samples. Instead, a range of established p53-wt containing cancer cells with isogenic p53-null controls was employed. The response of CD276 and PD-L1 to pharmacological or genetic perturbation in this range of cancer cell lines highlights the translatability of these pathways in different cell models. We used three p53-wt cancer cell models. HCT116 cells are one of the most widely used p53-wt containing cancer cells that also have a matched isogenic p53-null counterpart [33]. These cells are also very well characterized with respect to Nutlin-3 responses [8]. We used HCT116 cells in selected experiments when Nutlin-3 effects required p53-dependent validation, including (i) examination of the p53 dependence of CD276 and PD-L1 induction by Nutlin-3 using HCT116 p53-null cells as a control and (ii) examination of the Nutlin-3 response to changes in cell cycle parameters because HCT116 cells respond to Nutlin-3 through apoptosis [27, 39, 59].

A375 is a melanoma cell line model [48] used to study some p53-MDM2 responses [49, 60], although it is not so widely used since there are no published isogenic p53-null derivatives. In addition, since melanoma is a condition in which PD-L1 antibodies show therapeutic activity [61], in hindsight this model might be useful to determine whether PD-L1 is induced in a similar manner to that observed in HCT116 cells. Using this cell line, we were able to: (i) validate CD276 and MDM2 association by PLA; (ii) create a panel of new p53-null cells to validate p53 dependence in CD276 and PD-L1 induction by FACS; and (iii) use this new A375 p53-null cell panel to evaluate the effect of ATM. An important question to address was the p53 dependence of CD276 induction by Nutlin-3 and the elevated PD-L1 in A375 cells that have lost p53. By using newly constructed p53-null cell lines isolated from single cells by flow cytometry, we can estimate that the cell lines would have undergone approximately 30 cell divisions from the time of single cell cloning and analysis by immunoblotting and flow cytometry. The A375 p53-null cell lines recapitulate the trends observed using the HCT116 cell panels: (i) CD276 induction by Nutlin-3 is p53-dependent; (ii) PD-L1 induction by Nutlin-3 is p53-independent; and (iii) PD-L1 induction is elevated by ablation of the *TP53* gene. In the latter case, we also evaluated basal PD-L1 levels by flow cytometry in a range of p53-null cell clones, highlighting heterogeneity in PD-L1 basal levels that might reflect other genetic changes that occurred upon survival and growth of single cell lines or heterogeneity in the original A375 parental cell population. We can therefore have a degree of confidence in the response of CD276 and PD-L1 in relation to p53 status due to this newly created p53-null cell panel.

The final p53-wt cell model we used was A549, which was integrated as a final control to distinguish CD276 and PD-L1 responses to proteasome inhibitors. In examining the mechanism of CD276 or PD-L1 protein level changes by the proteasome pathway, we observed PD-L1 protein reduction by treatment with MG132. This is suggestive of an autophagic response. We used the A549 cell line model as this cell line exhibits constitutive autophagy as an oncogenic event [62]. This Atg5-null cell model having activated Ras and p53-wt has been recently used to establish a novel Atg5-signaling pathway in vivo [32]. Our data demonstrate striking upregulation of PD-L1 by loss of Atg5, together with enhanced suppression of T-cell proliferation. This contrasts with CD276, which shows no changes upon *ATG5* inactivation, suggesting again that CD276 and PD-L1 are under distinct genetic regulation. A549 cells were thus used to assess the effects of autophagic signaling changes at the PD-L1 or CD276 protein level and to highlight the specificity of CD276 and PD-L1 induction by Nutlin-3 only in p53-wt cell lines.

Prior to this study, the only well-established oncogenic pathway activated by Nutlin-3 was Notch; intracellular Notch receptor activation is controlled by MDM2-mediated monoubiquitination [24]. In this study, we validated a novel MDM2-interacting protein, CD276, which is an emerging pro-oncogenic receptor [63]. In addition, we demonstrated that the CD276 paralog PD-L1 is also induced by Nutlin-3. However, PD-L1 does not bind MDM2 and is elevated even further in cells without functional p53, or without functional Atg5. The reason why MDM2 would have evolved an interaction with CD276, but not with PD-L1, is unclear. The human CD276 and PD-L1 proteins have diverged significantly, with less than 50% identity, so it is difficult to define a protein interaction domain exploited by MDM2 that is selective for CD276. The p53-MDM2 axis evolved 500 million years prior to the advent of vertebrates [64]. P53 and MDM2 are also present in *Ciona intestinalis*, an organism which is a member of the subphylum Tunicata in the chordate phylum of marine invertebrates, considered the closest relatives to the vertebrates [65]. *Ciona intestinalis* harbors a gene encoding a protein with over 20% identity to human CD276 (but not PD-L1; data not shown). Therefore we could speculate that MDM2 has had sufficient geological time to evolve a protein-protein interaction with an ancient ancestor of the CD276 gene, under conditions in which the PD-L1 ancestral gene had not yet appeared in the biosphere.

From our findings, we conclude that CD276 and PD-L1 are under distinct genetic control, as summarized in Fig.8. The elevation of PD-L1 is a result of genetic ablation of the *ATG5* or *TP53* gene. We propose that, since Nutlin-3 can suppress p53-dependent autophagy [32, 66], either genetic loss of p53 (that reduces basal MDM2 protein) or targeting MDM2 using the ligand Nutlin-3 similarly attenuate the Atg5-signaling pathway leading to up-regulation of PD-L1. In contrast to PD-L1, neither p53 deletion nor Atg5 deletion up-regulates CD276. However, Nutlin-3 induces CD276 in a p53-dependent manner (Figs. 3 and 4). Since MDM2 associates with CD276 in Nutlin-3 treated cells (Fig. 2), we suggest that MDM2 itself is directly responsible for stabilization of CD276 protein. The majority of T-cell suppression is linked to PD-L1, and not CD276, since induction of PD-L in Atg5-null cells, and its attenuation by PD-L1 blocking antibodies, accounts for essentially all T-cell suppression. By contrast, siRNA mediated depletion of CD276 potentiates Nutlin-3 reduction in G1 and elevation in the G2 population of cells (Fig. 7). Together, these data suggest that, if Nutlin-3 co-induction of the PD-L1 and CD276 receptors occurs in vivo, then the former would mediate CD4+ T-cell suppression whilst the latter would impact on cell cycle equilibrium.

## Conclusion

The most compelling anti-cancer drugs to emerge in recent years are those that impact on immune checkpoint pathways [67]. The success of antibodies targeting cytotoxic T-lymphocyte-associated protein 4 (CTLA4), programmed cell death 1 (PD-1), or PD-L1 in stimulating immune cell mediated tumor rejection suggests that the immune system is poised for exploitation as a therapeutic target [66]. Nonetheless, only some patients respond to immune blockade therapies, and modifying determinants need to be identified. In this study, we performed assays to determine whether MDM2 perturbation had an impact on the oncogenic receptor PD-L1 and CD276. Both of these immune blockade paralog receptors are induced by Nutlin-3, although they are under different genetic control by p53 (Fig. 8). CD276 induction by Nutlin-3 is p53-dependent, whilst PD-L1 is elevated in p53-null or Atg5-null cells. One interpretation of these data showing that PD-L1 is elevated in p53-null cells is that Nutlin-3 creates a p53 hypomorphic phenotype because Nutlin-3 ‘inhibition’ of MDM2 is mimicked by the p53-null phenotype (e.g. p53-null cells produce lower MDM2 levels). Thus, it might be that CD276 induction by Nutlin-3 is p53-dependent and is mediated by direct binding of MDM2 to CD276, whilst PD-L1 is suppressed by a p53/MDM2-signaling event (Fig. 8). A recent report using HCT116 p53-wt and HCT116 p53-null cells highlighted that PD-L1 is regulated by p53-dependent induction of microRNA-34 (mir-34) targeting the 3’UTR determinant that regulates PD-L1 expression [68]. This is consistent with our data showing that p53-null cells produce higher levels of PD-L1. An interesting feature of these data is that, since the Nutlin-3 class of drugs can induce immune blockade receptors PD-L1 and CD276, this might produce an oncogenic effect of the drug in vivo by attenuating T-cell killing of tumor cells. Future studies can determine whether PD-L1 or PD-1 blocking antibodies, or CD276 antagonists, might augment the Nutlin class of drugs in the clinic. We would also suggest that by incorporating additional methodological assays in clinical trials that evaluate MDM2 targeted ligands, information can be acquired relating to pro-oncogenic off-target induction due to impacts on MDM2-dependent proteostasis.

## Supplementary information

**Additional file 1: Supplementary Fig. 1** Generating a melanoma cell line with a p53-null status using CRISPR mediated gene editing. The sequence of sgRNA targeting exon 5 of the p53 gene was 5′-CTGAGCAGCGCTCATGGTGGNGG-3′. The sgRNA was cloned into the pBT-U6-CAS9-2A-GFP expression vector to create pBT-U6-CAS9-2A-gp53-GFP. p53 gene editing in the A375 p53-wt melanoma cell line was performed as described before with minor alterations [35]. **a** The position and orientation of sgRNA targeting exon 5 of *TP53* sgRNA is indicated in blue, with PAM sequence in red. **b, c** Representative DNA sequences of the knockout p53 cell clone (E23). **b** p53 seq1 represents an out-of-frame deletion (dotted line) in one allele. **c** p53 seq2 represents in-frame insertion of cytosine (in red) in the other allele. **d** Western blotting of cell clones after X-irradiation that normally stabilizes p53-wt protein. p53 and GAPDH have apparent molecular weights on Western blots of 50 and 37 kDa, respectively. Clones with decreased levels or truncated forms of p53 are marked with an asterisk (A6, E1, E3, E18, E23, E26 and E28). **e** A375 cells X-irradiated (2.5 Gy) for indicated times. Detection of p53, p21^WAF1^ and GAPDH was found at 50, 20 and 37 kDa. **f** A375 p53-wt and A375 p53-null cells (clone E23) 4 h after X-irradiation. Detection of p53, p21^WAF1^ and GAPDH was performed.

**Additional file 2: Supplementary Fig. 2** Detection of Atg5 and α-tubulin in A549-Atg5-wt (wt), in A549-Atg5-wt processed using CRISPR to generate mutant A549-Atg5-null (null) and in an isolate of A549-atg5-null cells with re-introduced stably expressed green fluorescent protein (GFP)-Atg5 (resc). Atg5 and α-tubulin as a loading control have apparent molecular weights on Western blots of 56, 85 and 50 kDa, respectively.

**Additional file 3: Supplementary Fig. 3** FACS histograms showing CD276 and PD-L1 on HCT116 p53-wt, A549 Atg5-wt, A549 Atg5-null and A549 Atg-resc upon activation of autophagy by treatment with 10 μM chloroquine for the indicated times.

**Additional file 4: Supplementary Table 1** Summary of the MDM2 protein-protein interactions known to be regulated by Nutlin-3. Abbreviations: SPR (surface plasmon resonance), NPM (MDM2-nucleophosmin interaction), CypB (cyclophilin B), GRK2 (G-protein-coupled receptor kinase 2), GPR17 (G-protein-coupled receptor), DLD (dihydrolipoamide dehydrogenase), YFP (yellow fluorescent protein), SWATH (sequential window acquisition of all theoretical mass spectra).

## Data Availability

The data supporting the conclusions of this article are available from the corresponding author on reasonable request.

## Abbreviations

APC: Allophycocyanin
ATG5: Autophagy related 5
ATM: Ataxia-telangiectasia mutated
BSA: Bovine serum albumin
CD276: Cluster of differentiation 276
CD4+ T: CD4 positive T cells
CFSE: Carboxyfluorescein succinimidyl ester
CTLA4: T-lymphocyte-associated protein 4
cypB: Cyclophilin B
DLD: Dihydrolipoamide dehydrogenase
FACS: Fluorescence-activated cell sorting
FBS: Fetal bovine serum
FITC: Fluorescein isothiocyanate
GAPDH: Glyceraldehyde 3-phosphate dehydrogenase
GFP: Green fluorescent protein
HSP70: Heat shock protein 70
MDM2: Mouse double minute 2 homolog
Nut-3: Nutlin-3
MFI: Mean fluorescence intensity
Mir-34: microRNA-34
NPM: MDM2-nucleophosmin interaction
PBS: Phosphate buffered saline
PD-1: Programmed cell death 1
PD-L1: Programmed death-ligand 1
PLA: Proximity ligation assay
sgRNA: Single guide RNA
wt: Wild type
