# Supplementary figures and images for: The MDM2 ligand Nutlin-3 differentially alters expression of the immune blockade receptors PD-L1 and CD276
Source: Cell Mol Biol Lett. 2020 Aug 31;25:41. doi: 10.1186/s11658-020-00233-w (PMC7457494; doi:10.1186/s11658-020-00233-w)

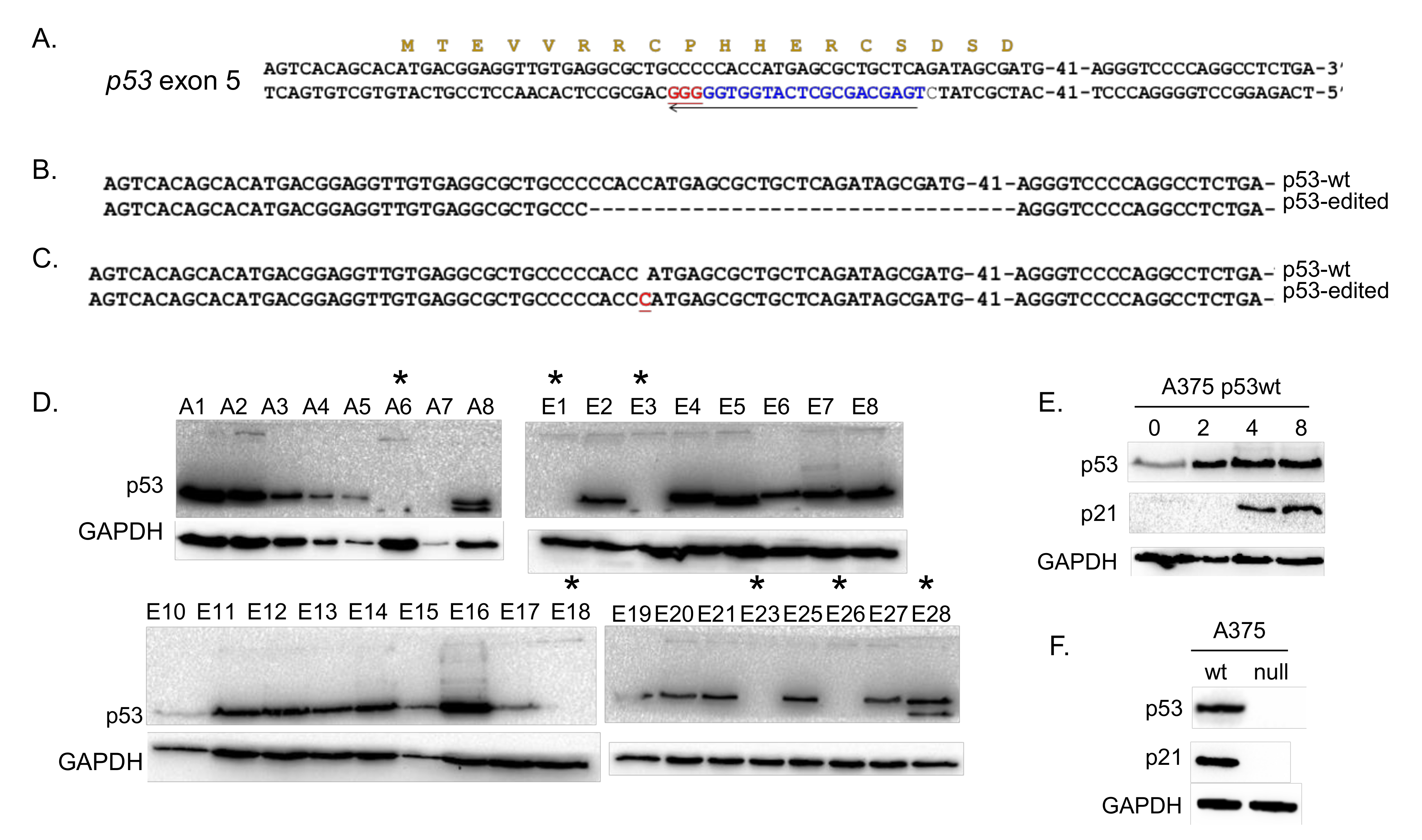

Supplement: Supplementary file 1 — Additional file 1: Supplementary Fig. 1 Generating a melanoma cell line with a p53-null status using CRISPR mediated gene editing. The sequence of sgRNA targeting exon 5 of the p53 gene was 5′-CTGAGCAGCGCTCATGGTGGNGG-3′. The sgRNA was cloned into the pBT-U6-CAS9-2A-GFP expression vector to create pBT-U6-CAS9-2A-gp53-GFP. p53 gene editing in the A375 p53-wt melanoma cell line was performed as described before with minor alterations [35]. a The position and orientation of sgRNA targeting exon 5 of TP53 sgRNA is indicated in blue, with PAM sequence in red. b, c Representative DNA sequences of the knockout p53 cell clone (E23). b p53 seq1 represents an out-of-frame deletion (dotted line) in one allele. c p53 seq2 represents in-frame insertion of cytosine (in red) in the other allele. d Western blotting of cell clones after X-irradiation that normally stabilizes p53-wt protein. p53 and GAPDH have apparent molecular weights on Western blots of 50 and 37 kDa, respectively. Clones with decreased levels or truncated forms of p53 are marked with an asterisk (A6, E1, E3, E18, E23, E26 and E28). e A375 cells X-irradiated (2.5 Gy) for indicated times. Detection of p53, p21WAF1 and GAPDH was found at 50, 20 and 37 kDa. f A375 p53-wt and A375 p53-null cells (clone E23) 4 h after X-irradiation. Detection of p53, p21WAF1 and GAPDH was performed. [file 11658_2020_233_MOESM1_ESM.tif]

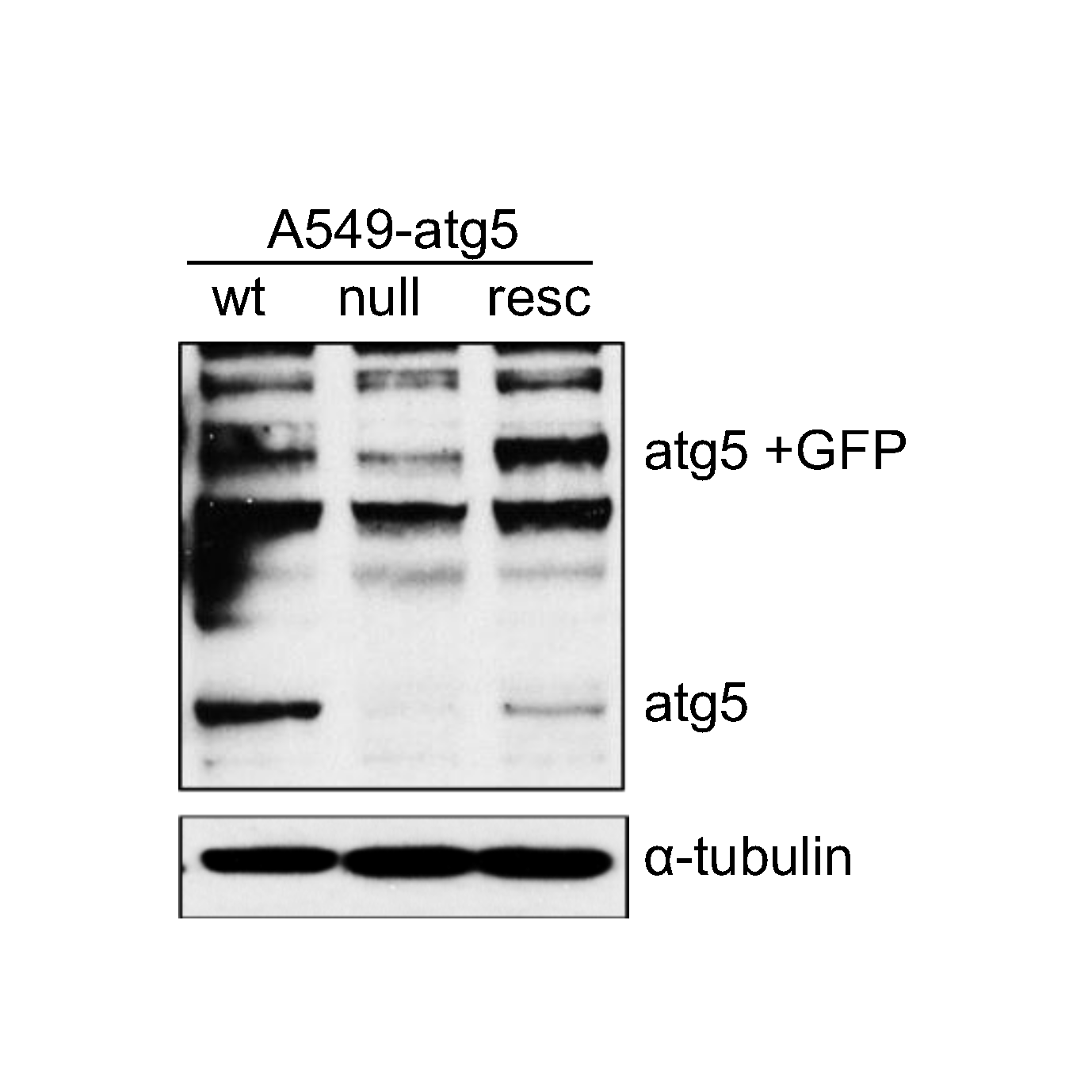

Supplement: Supplementary file 2 — Additional file 2: Supplementary Fig. 2 Detection of Atg5 and α-tubulin in A549-Atg5-wt (wt), in A549-Atg5-wt processed using CRISPR to generate mutant A549-Atg5-null (null) and in an isolate of A549-atg5-null cells with re-introduced stably expressed green fluorescent protein (GFP)-Atg5 (resc). Atg5 and α-tubulin as a loading control have apparent molecular weights on Western blots of 56, 85 and 50 kDa, respectively. [file 11658_2020_233_MOESM2_ESM.tif]

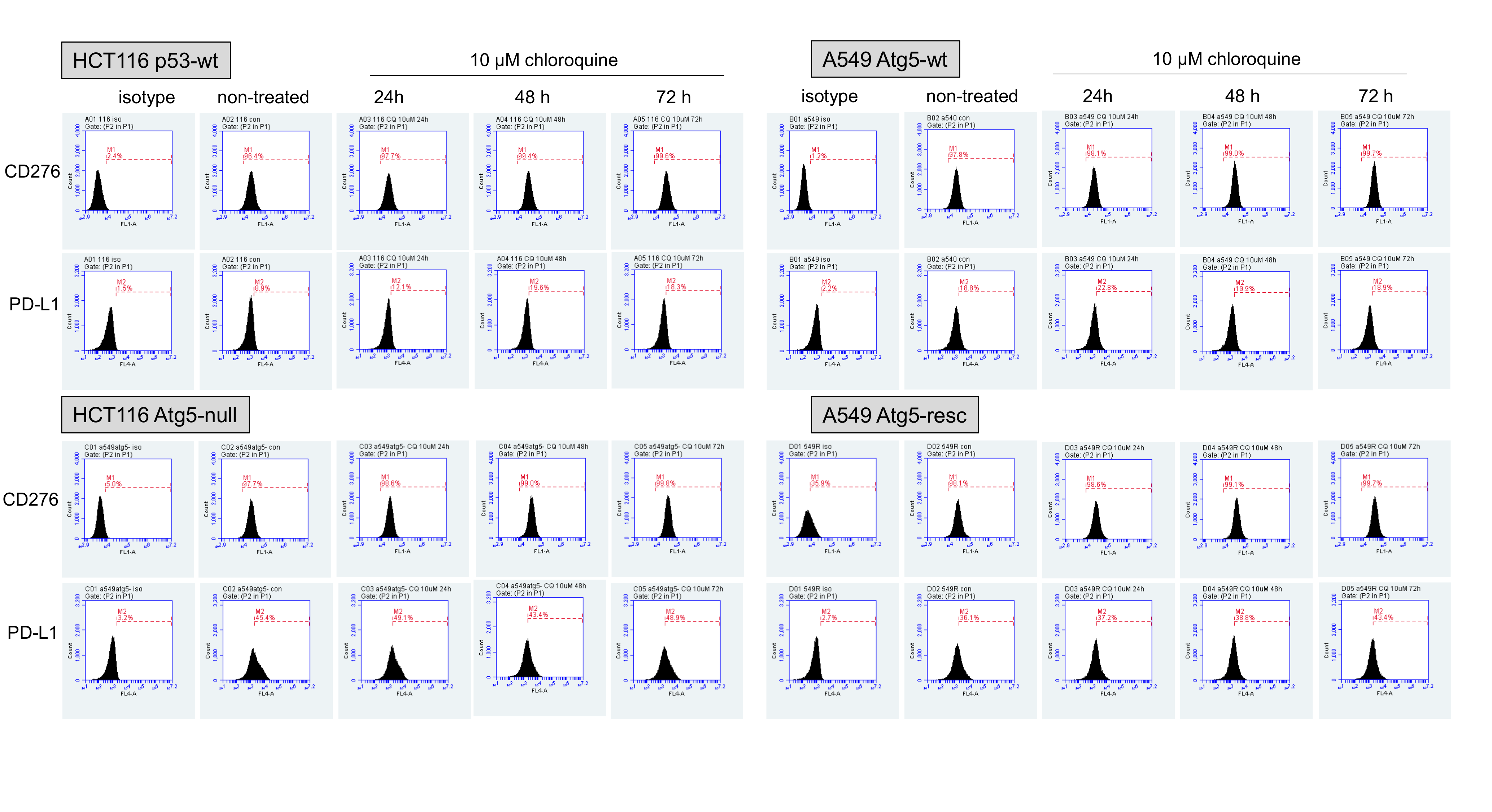

Supplement: Supplementary file 3 — Additional file 3: Supplementary Fig. 3 FACS histograms showing CD276 and PD-L1 on HCT116 p53-wt, A549 Atg5-wt, A549 Atg5-null and A549 Atg-resc upon activation of autophagy by treatment with 10 μM chloroquine for the indicated times. [file 11658_2020_233_MOESM3_ESM.tif]
