# Supplementary material for: The MDM2 ligand Nutlin-3 differentially alters expression of the immune blockade receptors PD-L1 and CD276
Source: Cell Mol Biol Lett. 2020 Aug 31;25:41. doi: 10.1186/s11658-020-00233-w (PMC7457494; doi:10.1186/s11658-020-00233-w)
Supplement: Supplementary file 4 — Additional file 4: Supplementary Table 1 Summary of the MDM2 protein-protein interactions known to be regulated by Nutlin-3. Abbreviations: SPR (surface plasmon resonance), NPM (MDM2-nucleophosmin interaction), CypB (cyclophilin B), GRK2 (G-protein-coupled receptor kinase 2), GPR17 (G-protein-coupled receptor), DLD (dihydrolipoamide dehydrogenase), YFP (yellow fluorescent protein), SWATH (sequential window acquisition of all theoretical mass spectra). [file 11658_2020_233_MOESM4_ESM.pdf]

Screen	Assay	Target	Impact of Nutlin-3	Reference
**MDM2 binding**	SPR	MDM2	*In vitro* disruption of MDM2-p53 protein complex	[8]
**Ubiquitination**	Activation of p53F19A ubiquitination by MDM2	P53	Stimulating of p53	[16]
**iTRAQ data dependent mass spectrometry**	Proteostasis	NPM	NPM de-oligomerization	[25]
**Linear motif screen**	Proteostasis	CypB	CypB induction	[26]
**Linear motif screen**	P53 homology	Notch	Notch induction	[24]
**Oligodendrocyte maturation**	ELISA	GRK2	GPR17 receptor suppression	[72]
**Chemotherapy synergy**	DNA repair	NBS1	Inhibition of DNA break repair	[73]
**SWATH data**	Proteostasis	DLD	Pyruvate dehydrogenase complex dissociation	[27]
**MDM2 binding**	YFP-p53	MDM2	Stabilization of YFP-p53 in cells	[74]
**Endogenous p53-MDM2 binding**	P53-ubiquitination	P53-Ub	In vivo induction of MDM2-p53 complex stimulates p53 mono-Ub on chromatin	[20]

[8] Vassilev LT, Vu BT, Graves B, Carvajal D, Podlaski F, Filipovic Z, Kong N, Kammlott U, Lukacs C, Klein C et al. In vivo activation of the p53 pathway by small-molecule antagonists of MDM2. Science.2004;303:844-848.

[16] Wallace M, Worrall E, Pettersson S, Hupp TR, Ball KL. Dual-site regulation of MDM2 E3-ubiquitin ligase activity. Mol Cell.2006;23:251-263.

[25] Nicholson J, Neelagandan K, Huart AS, Ball K, Molloy MP, Hupp T. An iTRAQ Proteomics Screen Reveals the Effects of the MDM2 Binding Ligand Nutlin-3 on Cellular Proteostasis. J Proteome Res.2012;11:5464-5478.

[26] Nicholson J, Scherl A, Way L, Blackburn EA, Walkinshaw MD, Ball KL, Hupp TR. A systems wide mass spectrometric based linear motif screen to identify dominant in-vivo interacting proteins for the ubiquitin ligase MDM2. Cell Signal.2014;26:1243-1257.

[24] Pettersson S, Sczaniecka M, McLaren L, Russell F, Gladstone K, Hupp T, Wallace M. Non-degradative ubiquitination of the Notch1 receptor by the E3 ligase IVIDM2 activates the Notch signalling pathway. Biochem J.2013;450:523-536.

[72] Fumagalli M, Bonfanti E, Daniele S, Zappelli E, Lecca D, Martini C, Trincavelli ML, Abbracchio MP. The Ubiquitin Ligase Mdm2 Controls Oligodendrocyte Maturation by Intertwining mTOR with G Protein-Coupled Receptor Kinase 2 in the Regulation of GPR17 Receptor Desensitization. Glia. 2015;63:2327-2339.

[73] Carrillo AM, Hicks M, Khabele D, Eischen CM. Pharmacologically Increasing Mdm2 Inhibits DNA Repair and Cooperates with Genotoxic Agents to Kill p53-Inactivated Ovarian Cancer Cells. Mol Cancer Res. 2015;13:1197-1205.

[27] Way L, Faktor J, Dvorakova P, Nicholson J, Vojtesek B, Graham D, Ball KL, Hupp T. Rearrangement of mitochondrial pyruvate dehydrogenase subunit dihydrolipoamide dehydrogenase protein-protein interactions by the MDM2 ligand nutlin-3. Proteomics. 2016;16:2327-2344.

[74] Stewart-Ornstein J, Lahav G. p53 dynamics in response to DNA damage vary across cell lines and are shaped by efficiency of DNA repair and activity of the kinase ATM. Sci Signal. 2017;10: (476):eaah6671.

[20] Landre V, Revi B, Mir MG, Verma C, Hupp TR, Gilbert N, Ball KL. Regulation of transcriptional activators by DNA-binding domain ubiquitination. Cell Death Differ. 2017;24:903-916.
